# Immune Checkpoint Inhibitor-Induced Colitis Presenting As Lymphocytic Colitis

**DOI:** 10.7759/cureus.18085

**Published:** 2021-09-18

**Authors:** Jaffar Khan, Terrence Katona

**Affiliations:** 1 Pathology and Laboratory Medicine, Indiana University School of Medicine, Indianapolis, USA

**Keywords:** cpis, lymphocytic colitis, immunotherapy, diarrhea, check pointe inhibitor

## Abstract

Checkpoint inhibitors (CPIs) are a new class of drugs that have changed the treatment and prognosis of several malignancies, even in their advanced stages. These drugs have increased patient survival rates. CPIs stimulate the immune system and include cytotoxic T-lymphocyte antigen-4 inhibitors (ipilimumab), programmed cell death inhibitors such as pembrolizumab, nivolumab, and avelumab, and programmed cell death protein ligand-1 inhibitors such as atezolizumab. Herein, we present a case of CPI-induced colitis in a 45-year-old woman with a history of melanoma. The melanoma was BRAF-positive with a V600 mutation. She had metastasis to the brain and the right 10th rib, which underwent surgery and radiation treatment, respectively. She was treated with nivolumab and denosumab. The patient presented with chronic watery diarrhea. Biopsy revealed lymphocytic colitis-like changes in the colon and terminal ileum. Thus, given the history of CPIs, a diagnosis of CPIS-induced colitis was made.

## Introduction

Immune checkpoint inhibitors (CPIs) are a class of drugs that stimulate the immune system to achieve an antitumor response. They block inhibitory signals to cytotoxic T-lymphocytes, thus enhancing immune function [1]. They have changed the prognosis of melanoma and other cancers [2]. The targets of CPIs are cytotoxic T-lymphocyte-associated antigen-4 and programmed cell death 1 receptor [3]. Despite their role in treatment, CPIs can cause immune-related adverse events such as rash, fatigue, etc. in other organs, and diarrhea in the gastrointestinal tract [4]. These blocking antibodies target the immune checkpoint molecules CTLA-4 and PD-1 and have been approved for the treatment of metastatic melanoma in 2011, non-small-cell lung cancers, and metastatic renal cancer [5].

## Case presentation

**Figure 1:**
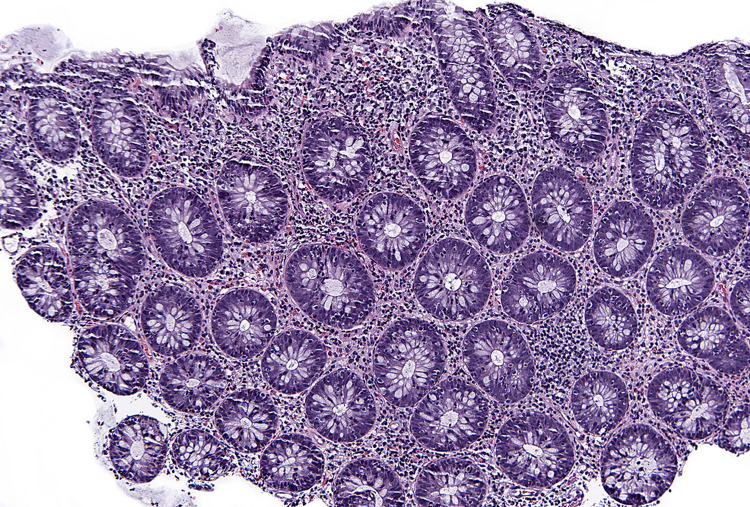
Microscopic examination of the colon showing increased lymphocytes in the surface epithelium and within the crypts. Hematoxylin and eosin staining (H&E) (10x).

**Figure 2:**
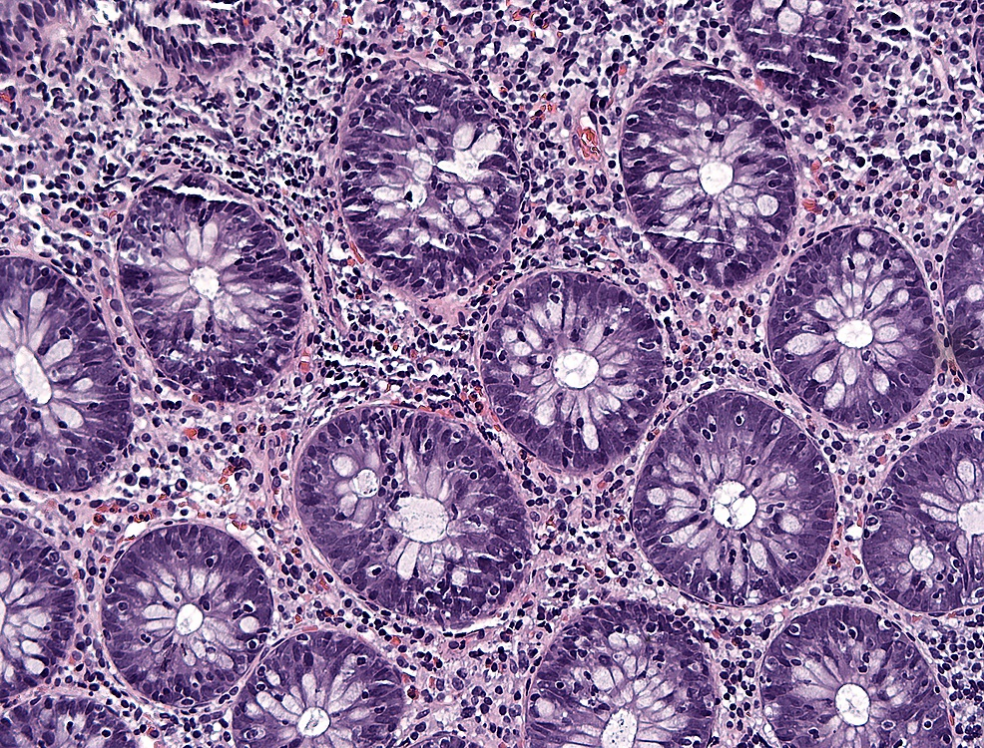
The colonic mucosa showing increased lymphocytes within the crypt epithelium. H&E (20x).

A previously healthy 45-year presented with a skin lesion on her back. A biopsy of the lesion on her right upper back revealed melanoma in 2017. The patient underwent tumor resection. Later, another lesion was resected from the hip area, which revealed early pre-invasive melanoma over the left lateral anterior pelvis. At that time, the physicians thought the melanoma had not spread; unfortunately, in July 2020, imaging revealed brain and lung lesions, and the tumor had metastasized to the brain and lungs. The melanoma was BRAF-positive, with a V600 mutation. She underwent left frontal lesion resection in August 2020, followed by radiation therapy. In January 2021, she had metastasis to the right 10th rib, which received radiation treatment. The patient was treated with nivolumab (an immune CPI). The patient presented with chronic watery diarrhea 11 months after receiving treatment. Biopsy of the terminal ilium and random colon was performed to reveal colonic mucosa with increased lymphocytes within the surface epithelium and in some crypt epithelium (Figure 1). The crypts had increased lymphocytes with an overall pattern resembling lymphocytic-like colitis (Figure 2). The differential diagnosis was ANTI-PD-L1 immunotherapy-induced colitis.

## Discussion

CPIs are now used to treat a variety of tumors, including melanoma, non-small cell carcinoma of the lung, and renal cell carcinoma. Their increased use will also increase the adverse immune-related reactions associated with these drugs [4]. The CPIs CTLA-4, PD-1, and PD-L1 receptors play an important role in T-cell immunity and downregulate receptors. Blocking these checkpoints results in increased proliferation of effector T-cells, thus increasing the immune response [6]. Ipilimumab inhibits CTLA-4, thus enabling cytotoxic T-lymphocytes to exert their cytotoxic effects on tumor cells [6,7]. As the number of cancer cases increases over time, increasing efforts are being made to treat them. CPIs are a family of drugs used to treat a variety of cancers. The downside of these drugs is an immune-induced adverse effect due to abnormal self-tolerance. The side effects in the liver may result in hepatitis and colitis in the gastrointestinal tract, resulting in diarrhea [6].

There is not much information in the current literature about the mechanism of immune-related injury caused by CPIs in the GI tract. Endoscopic examinations are usually normal or have mild lesions. The diagnosis was made based on the histopathological changes observed on the biopsy. Microscopic colitis has two forms: collagenous and lymphocytic colitis. Both are characterized by epithelial alterations with inflammatory infiltrates in the lamina propria with mixed immune cells. Lymphocytic colitis increases the number of intraepithelial lymphocytes [8].

CPI-induced colitis shows active focal colitis-like changes with patchy crypt abscesses to diffuse mucosal inflammation on histological examination [9]. Biopsies usually reveal features of acute colitis with increased cellularity of the lamina propria and inflammatory cells such as mononuclear cells and other changes, including intraepithelial neutrophilic infiltrates and crypt abscesses, along with an increased number of apoptotic cells in crypts [10]. Histological findings can precede the onset of colitis and diarrhea. A study of patients who underwent colonoscopy one-two weeks after induction with ipilimumab showed colitis-like changes followed by clinical symptoms [11].

CPI-induced colitis is most commonly observed in patients receiving ipilimumab [12]. Endoscopy usually shows diffuse ulceration and edema, and diffuse enteritis can be observed throughout the colon [13]. Biopsy of the affected colon usually reveals colonic mucosa with mixed lymphocytic and neutrophilic infiltrate with apoptotic mucosal epithelial cells and crypt abscesses. However, the structure of the epithelium was preserved in comparison to that of inflammatory bowel disease. Thus, even if there is no macroscopic evidence of the disease, biopsy should be considered in the presence of an appropriate clinical scenario. The presence of active inflammation and plasmacytosis, along with the presence of large(>1 cm) ulcerations, is predictive of a requirement for biological therapies [14]. Corticosteroids are the recommended first-line treatment for CPI-induced colitis. The combination of corticosteroids with infliximab is associated with a shorter recovery time in CPI-induced high-grade colitis [14].

## Conclusions

The presentation of our case in a patient with a history of CPIs is a good example of CPI-induced colitis presenting with lymphocytic colitis. Therefore, complete patient history and the entire scenario are essential for pathologists to make a diagnosis. Given the rise in cancer cases, we expect more use of immune CPIs in the future; thus, pathologists need to be aware of the histopathological findings and variants for the correct diagnosis of CPI-induced colitis.
